# Supplementary material for: Genomic Characterization of Mycobacterium leprae to Explore Transmission Patterns Identifies New Subtype in Bangladesh
Source: Front Microbiol. 2020 Jun 16;11:1220. doi: 10.3389/fmicb.2020.01220 (PMC7308449; doi:10.3389/fmicb.2020.01220)
Supplement: FIGURE S1 — Samples analyzed by whole genome sequencing. [file Data_Sheet_3.docx]

**Supplementary Material**

**Genomic characterization of *Mycobacterium leprae* to explore** **transmission patterns identifies new subtype in Bangladesh**

**Maria Tió-Coma, Charlotte Avanzi,** **Els M. Verhard, Louise Pierneef, Anouk van Hooij,**

**Andrej Benjak, Johan Chandra Roy, Marufa Khatun, Khorshed Alam, Paul Corstjens,**

**Stewart T. Cole, Jan Hendrik Richardus, and Annemieke Geluk**

# Supplementary Tables

**Table S1. Cohort characterization.**

| **Group** | **Subjects** | **Gender** | **Age** | **RJ Classification** | **BI** |
| --- | --- | --- | --- | --- | --- |
| MB patients BI 2-6* | 33 | 18% Female  82% Male | 34 | 17 LL  15 BL  1 BT | 14 BI-6  3 BI-5  5 BI-6  6 BI-5  4 BI-4  1 BI-2 |
| PB/MB patients BI 0 | 27 | 63% Female  37% Male | 31 | 24 BT  2 TT  1 UD | 24 BI-0  3 UD |
| HHC | 250 | 52% Female  48% Male | 30 | - | - |

Group, number of subjects, percentage of female and male, median of age, Ridley-Jopling classifications and BI of patients are shown. *31 were index cases of the study. UD: Undetermined.

**Table S2**. **Primers and probes used in the study.**

| **Primer** | **Test** | **Sequence 5**'**-3'** | **Amplicon size (bp)** |
| --- | --- | --- | --- |
| RLEP LP1 F | PCR | tgcatgtcatggccttgagg | 129 |
| RLEP LP2 R | PCR | caccgataccagcggcagaa |  |
| RLEP qPCR F | qPCR | gcagcagtatcgtgttagtgaa | 73 |
| RLEP qPCR R | qPCR | cgctagaaggttgccgtat |  |
| RLEP Probe FAM | qPCR | cgccgacggccggatcatcga |  |
| SNP 14676 F (Locus 1) | PCR+seq | aatggaatgctggtgagagc | 194 |
| SNP 14676 R (Locus 1) | PCR+seq | caatgcatgctagccttaatga |  |
| SNP 1642879 F (Locus 2) | PCR+seq | ttgaatgcgaccaaacgtactttctg | 122 |
| SNP 1642879 R (Locus 2) | PCR+seq | taccaccggatcatggaaccgtc |  |
| SNP 2935685 F (Locus 3) | PCR+seq | atctggtccgggtaggaatc | 180 |
| SNP 2935685 R (Locus 3) | PCR+seq | accggtgagcgcactaag |  |
| SNP 8453 F (SNP1A) | PCR+seq | ggtctgcggacaagttggta | 211 |
| SNP 8453 R (SNP1A) | PCR+seq | caatagcgctcagacacgac |  |
| SNP 313361 F (SNP1B) | PCR+seq | caccggagacaaagctgat | 200 |
| SNP 313361 R (SNP1B) | PCR+seq | ctcggagaccaaacttctcg |  |
| SNP 61425 F (SNP1C) | PCR+seq | tcgtcaagccgaaagagttt | 243 |
| SNP 61425 R (SNP1C) | PCR+seq | ccagaacaccgagggaataa |  |
| SNP 889499 F (SNP1B-Bangladesh) | PCR+seq | cagctcggaaatccactctc | 199 |
| SNP 889499 F (SNP1B-Bangladesh) | PCR+seq | cgtcttcgacaccttgacct |  |
| *rpoB* F | PCR+seq | gtcgaggcgatcacgccgca | 279 |
| *rpoB* R | PCR+seq | cgacaatgaaccgatcagac |  |
| *folP1* F2 | PCR+seq | aactgatgctgcttctcgtg | 287 |
| *folP1* R2 | PCR+seq | ccctgtgctgcaagttcttt |  |
| *gyrA* F | PCR+seq | atggtctcaaaccggtacatc | 225 |
| *gyrA* R | PCR+seq | tacccggcgaaccgaaattg |  |

Forward (F) and reverse (R) primers and probes with dye used for RLEP PCR (Donoghue et al., 2001) and qPCR (Martinez et al., 2009), genotyping (Monot et al., 2009; Truman et al., 2011) and antimicrobial resistance (WHO, 2017). Test column indicates if the primers were used for PCR, qPCR or PCR and Sanger sequencing (PCR+seq). SNP 889499 to define new genotype 1B-Bangladesh changes from C to T in subtype 1B-Bangladesh.

**Table S3. *M. leprae* strain-specific SNPs.**

| **Subject ID** | **Position in the genome** | **Wild type** | **Mutation** | **Gene** | **Codon change** | **Amino acid change** |
| --- | --- | --- | --- | --- | --- | --- |
| H04I | 1081085 | C | T | *murD* | 865C>T | Leu289Phe |
|  | 1890100 | C | T | *cobB**** | 416C>T | - |
| H09I | 1693181 | A | G | *argR* | 82A>G | Thr28Ala |
|  | 5351 | C | T | *gyrB* | 123C>T | Leu41Leu |
| H10I | 1898238 | T | C | *ML1572* | 115T>C | Tyr39His |
|  | 1964656 | C | T | *ML1632* | 1253C>T | Pro418Leu |
| H15I | 34563 | G | A | *ML0029* | 76G>A | Val26Ile |
|  | 157111 | C | G | *ML0116* | 819C>G | His273Gln |
| H20I | 2391490 | A | G | *ctpB* | 2080A>G | Ile694Val |
|  | 2861093 | G | T | *ML2389**** | 221G>T | - |
| H23I | 160576 | T | C | *lipE* | 1155T>C | Ser385Ser |
|  | 354948 | C | G | *ML0271* | 273C>G | Val91Val |
| H24I | 1024176 | G | A | *ML0861* | 538G>A | Asp180Asn |
|  | 2022304 | C | A | *ddlA* | 670C>A | Leu224Met |
| H11I | 1157640 | A | G | *ML0990* | 360A>G | Ile120Met |
|  | 1802381 | C | G | *ML1495* | 434C>G | Pro145Arg |
| H18I | 3109974 | C | T | *bglS* | 211C>T | pseudogene |
|  | 1020650 | G | A | *lipA* | 764G>A | Arg255His |
| H17I | 856547 | G | T | *lhr* | 3267G>T | pseudogene |
|  | 2029182 | G | A | *ML1683* | 138G>A | Gly46Gly |
| H08I | 1182540 | A | G | *ML1012* | 429A>G | pseudogene |
|  | 1944088 | G | A | *ffh* | 1279G>A | Ala427Thr |
| H26I | 2094131 | C | T | *ML1733* | 147C>T | Arg49Arg |
|  | 3102710 | C | T | *ML2597* | 384C>T | Ala128Ala |
| H27I | 404990 | G | A | *ML0316* | 284G>A | Gly95Glu |
|  | 1368618 | C | T | *ML1171* | 127C>T | Leu43Phe |
| H29I | 2691282 | C | G | *ML2267* | 1097C>G | pseudogene |
|  | 3004830 | G | A | *ML2522* | 417G>A | Val139Val |
| H30I | 1140222 | C | T | *ML0971* | 170C>T | pseudogene |
|  | 3048182 | C | T | *nirB* | 406C>T | pseudogene |
| H05I | 52981 | C | T | *ML0042* | 989C>T | Ala330Val |
|  | 679359 | C | A | *uvrC* | 490C>A | His164Asn |
| H06I | 179474 | G | T | *ML0133* | 219G>T | Leu73Phe |
|  | 3109073 | G | A | *ML2604* | 628G>A | Ala210Thr |
| H07I | 1288026 | C | T | *ML1113* | 1601C>T | Ala534Val |
|  | 2625366 | C | G | *cpsY* | 1043C>G | Ala348Gly |
| H12I | 1747440 | T | C | *ML1452* | 122T>C | Val41Ala |
|  | 3254510 | A | G | *ML2700* | 1454A>G | His485Arg |
| H14I | 961300 | C | T | *rhlE* | 452G>A | Arg151Gln |
|  | 1417446 | C | G | *lspA* | 418C>G | Leu140Val |

List of mutated genes used to build strain specific primers (two SNPs per strain). Each mutation is unique to the strain analyzed compared to 259 other *M. leprae* genomes. *** indicate pseudogenes.

**Table S4**. **PCR primers for *M. leprae* strain-specific SNPs*.***

| **Subject ID** | **Position in the genome** | **Genes** | **Primer F (5’-3’)** | **Primer R (5’-3’)** | **Amplicon size (bp)** |
| --- | --- | --- | --- | --- | --- |
| H04I | 1081085 | *murD* | aatgccggttacgtcgatac | cagcaacggctaccacct | 164 |
|  | 1890100 | *cobB* | gtccactgcacacgtaatcg | acaccgacctgctcagatg | 207 |
| H09I | 1693181 | *argR* | cgcttcgaagaccacacc | gagctcttcgaggtctcgtg | 166 |
|  | 5351 | *gyrB* | gaaggcccaagacgaatatg | tcgaataaccgcacatcaac | 195 |
| H10I | 1898238 | *ml1572* | ttgacctacgcagcatatcg | agtcaggtgggtcagcaatc | 216 |
|  | 1964656 | *ml1632* | cactacaccaacgaccagga | gtggagaccacgacaacctt | 242 |
| H15I | 34563 | *ml0029* | gcggccatcgtcatacttta | atggtcaacaccgaggacat | 168 |
|  | 157111 | *ml0116* | attcacctcggtcccctatg | gaaaaggctgacgacgatcc | 193 |
| H20I | 2391490 | *ctpB* | ttgtagttccgacgacgatg | agcgcaaaacgtcgataact | 169 |
|  | 2861093 | *ml2389* | gccataatcgatcactggtgt | ggtcgatgtagggatgtgct | 172 |
| H23I | 160576 | *lipE* | ggattcatcttgggaacgaa | gcggtgatggtgttcattag | 197 |
|  | 354948 | *ml0271* | tcttaccggaacggctattg | agatcagctcaacgacagca | 218 |
| H24I | 1024176 | *ml0861* | tggctcgtctatcggtatca | gcgtcgtcgagatatttggt | 249 |
|  | 2022304 | *ddlA* | agatcggagactcggttcag | acctatccgtgccaactcac | 158 |
| H11I | 1157640 | *ml0990* | ctggtattcggtgtcggttt | agccatccagcagtgtcttt | 307 |
|  | 1802381 | *ml1495* | gtcagcgcacccatttatct | cgttcatcccagcttcaaat | 358 |
| H18I | 3109974 | *bglS* | gcgacgatgttgttgatgac | tgatgctgtatgccgttgat | 365 |
|  | 1020650 | *lipA* | gcctgctttcacctaccaac | gatacgatgaccgcaccag | 305 |
| H17I | 856547 | *lhr* | cagcgttcctgtcatcttca | cactcgacggattacctcgt | 466 |
|  | 2029182 | *ml1683* | caaagcagagctcattgacg | gcctttttagccggaacttt | 465 |
| H08I | 1182540 | *ml1012* | cgattctcggtgaagaccat | ggtgactttctcgtcgaagc | 358 |
|  | 1944088 | *ffh* | tcgatgacaaacagctcgac | cctgggaacttcagtttgga | 442 |
| H26I | 2094131 | *ml1733* | ggtatttgacgtggggagaa | ccgagtcggtagctaagtcg | 317 |
|  | 3102710 | *ml2597* | gcgagggattactgcttgtc | acctggaggaggatctcgtt | 316 |
| H27I | 404990 | *ml0316* | ccgaatactgggtgttgctt | aatgccgagttgtacgcttc | 468 |
|  | 1368618 | *ml1171* | cggagaaaaacctgggctat | cgccaaagatccataccatc | 327 |
| H29I | 2691282 | *ml2267* | ggccactaacctgccattta | tgttgctcagtggttcttcg | 347 |
|  | 3004830 | *ml2522* | tgggccaccttaatacttgc | tttgcaggttctcgctttct | 424 |
| H30I | 1140222 | *ml0971* | tggtcttcggtatcctcgtc | ttcgcgttactgcatagtcg | 454 |
|  | 3048182 | *nirB* | ctggtgttgcctagcaatga | gtgggtttgcaaccaaaact | 358 |
| H05I | 52981 | *ml0042* | tctgtggacacagcttcgag | cactgtctcgccaacgagta | 356 |
|  | 679359 | *uvrC* | aggtcgagtggacagtggtc | gttccaagtcacgggcatac | 450 |
| H06I | 179474 | *ML0133* | gggaagaaatccgaaagctc | ccataatctcgccgatagga | 321 |
|  | 3109073 | *ml2604* | tgcggacgtatgtacagctc | gcagctcagtgctgttgtct | 498 |
| H07I | 1288026 | *ml1113* | aaacattgcctacggacgac | tggaagatggtgtgcgataa | 313 |
|  | 2625366 | *cpsY* | ggcattgccaacctacaact | tgaggcctgtgtacgaagtg | 500 |
| H12I | 1747440 | *ml1452* | ggcagacggacttcaacct | tcatgacagtcaccccactc | 412 |
|  | 3254510 | *ml2700* | cgatcgtgatcattgtcgtc | atctggcacagtcgctttct | 482 |
| H14I | 961300 | *rhlE* | aaaatacctgaccgctgacg | atgggttggtccatgaatgt | 342 |
|  | 1417446 | *lspA* | gacacggtgacctggacttt | atgtcgaactcggctttacg | 377 |

Index case (Subject ID) with the *M. leprae* strain where the mutation was identified*.*

Table S5. WGS results.

| **Subject ID** | **Sample ID** | **Sample type** | **RLEP Ct** | **Overall alignment rate** | **Input reads** | **Mean Coverage** | **Standard deviation Coverage** | **Genotype** |
| --- | --- | --- | --- | --- | --- | --- | --- | --- |
| H09I | RB001 | SSS | 17.90 | 88.70 | 37876264 | 118.8 | 18.1 | 1B-Bangladesh |
| H24I | RB074 | SSS | 16.81 | 0.93 | 13530098 | 102.6 | 27.4 | 1B-Bangladesh |
| H07I | RB188 | SSS | 19.17 | 88.27 | 10622218 | 92.0 | 26.5 | 1D-*esxA* |
| H20I | RB069 | SSS | 18.94 | 0.75 | 21851287 | 89.6 | 21.7 | 1B-Bangladesh |
| H23I | RB073 | SSS | 16.25 | 0.89 | 9286514 | 86.1 | 29.0 | 1A |
| H08I | RB180 | SSS | 21.65 | 90.22 | 13937851 | 84.3 | 20.1 | 1D |
| H09C02 | RB003 | SSS | 21.63 | 0.56 | 23437425 | 46.6 | 13.6 | 1B-Bangladesh |
| H27I | RB182 | SSS | 19.04 | 52.87 | 5466606 | 45.8 | 19.3 | 1A |
| H26I | RB181 | SSS | 22.55 | 66.86 | 7136091 | 41.5 | 13.4 | 1D |
| H05I | RB186 | SSS | 23.19 | 96.15 | 3886798 | 33.8 | 11.0 | 1D |
| H23I | RN084 | Nasal swab | 20.06 | 0.10 | 21244851 | 30.8 | 15.5 | 1A |
| H29I | RB184 | SSS | 18.40 | 87.61 | 1812203 | 29.7 | 14.7 | 1D |
| H15I | RN059 | Nasal swab | 21.15 | 0.18 | 8947418 | 23.8 | 12.8 | 1D-*esxA* |
| H09I | RN001 | Nasal swab | 21.21 | 0.27 | 10293926 | 22.9 | 10.9 | 1B-Bangladesh |
| H10I | RN022 | Nasal swab | 23.86 | 0.09 | 58961720 | 19.1 | 10.5 | 1D |
| H14I | RB063 | SSS | 23.05 | 0.60 | 6035989 | 18.3 | 8.0 | 1A |
| H15I | RB048 | SSS | 23.25 | 0.69 | 14209772 | 16.4 | 10.0 | 1D-*esxA* |
| H24I | RN095 | Nasal swab | 21.24 | 0.02 | 86248391 | 16.4 | 9.6 | 1B-Bangladesh |
| H11I | RB041 | SSS | 23.13 | 74.65 | 1583694 | 15.4 | 7.1 | 1D |
| H10I | RB053 | SSS | 20.73 | 0.64 | 42419599 | 14.8 | 10.7 | 1D |
| H04I | RB066 | SSS | 23.71 | 0.92 | 45440398 | 14.4 | 11.2 | 1D |
| H12I | RB022 | SSS | 21.76 | 71.59 | 1023739 | 12.3 | 6.8 | 1A |
| H20I | RN165 | Nasal swab | 24.20 | 0.04 | 49770933 | 12.3 | 8.1 | 1B-Bangladesh |
| H06I | RB187 | SSS | 25.35 | 95.65 | 5938249 | 12.1 | 7.4 | 1D |
| H17I | RB067 | SSS | 22.96 | 93.36 | 5924859 | 11.3 | 6.5 | 1D |
| H30I | RB185 | SSS | 26.21 | 36.75 | 4508785 | 7.6 | 5.1 | 1A |
| H18I | RB065 | SSS | 21.21 | 78.72 | 8185166 | 6.1 | 5.9 | 1D-*esxA* |
| H16I | RN048 | Nasal swab | 23.27 | 5.42 | 3922066 | 4.2 | 3.3 |  |
| H22I | RN070 | Nasal swab | 26.70 | 0.02 | 25558536 | 1.9 | 4.2 |  |
| H21I | RB071 | SSS | 22.67 | 41.12 | 225851 | 1.8 | 1.6 |  |
| H13I | RB062 | SSS | 27.84 | 1.42 | 7741019 | 1.1 | 1.4 |  |
| H09C02 | RN003 | Nasal swab | 28.44 | 0.03 | 45483192 | 0.8 | 4.0 |  |
| H04I | RN180 | Nasal swab | 26.27 | 0.01 | 20827629 | 0.8 | 3.7 |  |
| H15C01 | RN060 | Nasal swab | 31.90 | 0.04 | 50724722 | 0.4 | 4.6 |  |
| H03I | RN131 | Nasal swab | 28.58 | 0.00 | 38908767 | 0.3 | 2.9 |  |
| H15C02 | RN061 | Nasal swab | 31.51 | 0.02 | 38484288 | 0.3 | 4.1 |  |
| H25I | RN190 | Nasal swab | 29.25 | 2.33 | 5882069 | 0.3 | 3.8 |  |
| H13C03 | RN085 | Nasal swab | 33.84 | 0.08 | 9083592 | 0.2 | 4.0 |  |
| H31I | RN186 | Nasal swab | 20.70 | 0.27 | 5374729 | 0.2 | 1.4 |  |
| H24C06 | RN098 | Nasal swab | 37.08 | 0.00 | 7876102 | 0.2 | 2.2 |  |
| H23C02 | RN089 | Nasal swab | 35.57 | 0.02 | 7052993 | 0.2 | 3.8 |  |
| H15C04 | RN062 | Nasal swab | 35.69 | 0.13 | 9975494 | 0.2 | 3.9 |  |
| H24C01 | RN091 | Nasal swab | 34.02 | 0.02 | 8358917 | 0.2 | 4.0 |  |
| H22C04 | RN080 | Nasal swab | 35.97 | 0.01 | 7236189 | 0.2 | 3.7 |  |
| H19I | RN182 | Nasal swab | 33.66 | 0.71 | 6722761 | 0.1 | 3.3 |  |
| H14I | RN065 | Nasal swab | 35.83 | 0.01 | 7248820 | 0.1 | 3.3 |  |
| H04C04 | RN155 | Nasal swab | 36.45 | 0.03 | 11927998 | 0.1 | 2.8 |  |
| H24C02 | RN093 | Nasal swab | 32.57 | 0.00 | 5643286 | 0.1 | 2.5 |  |
| H24C05 | RB108 | SSS | 37.14 | 0.02 | 9180110 | 0.1 | 1.5 |  |
| H22C03 | RN079 | Nasal swab | 36.74 | 0.00 | 10138274 | 0.1 | 2.4 |  |
| H14C01 | RB076 | SSS | 35.46 | 0.00 | 5344261 | 0.0 | 0.8 |  |

Data for the 51 samples sequenced according to the Ct obtained by qPCR. Coverage ≥5 was considered successful and data was used for further analysis. In the column Mean Coverage a color gradient from green to red represent maximum to minim coverage values.

# Supplementary figures

**
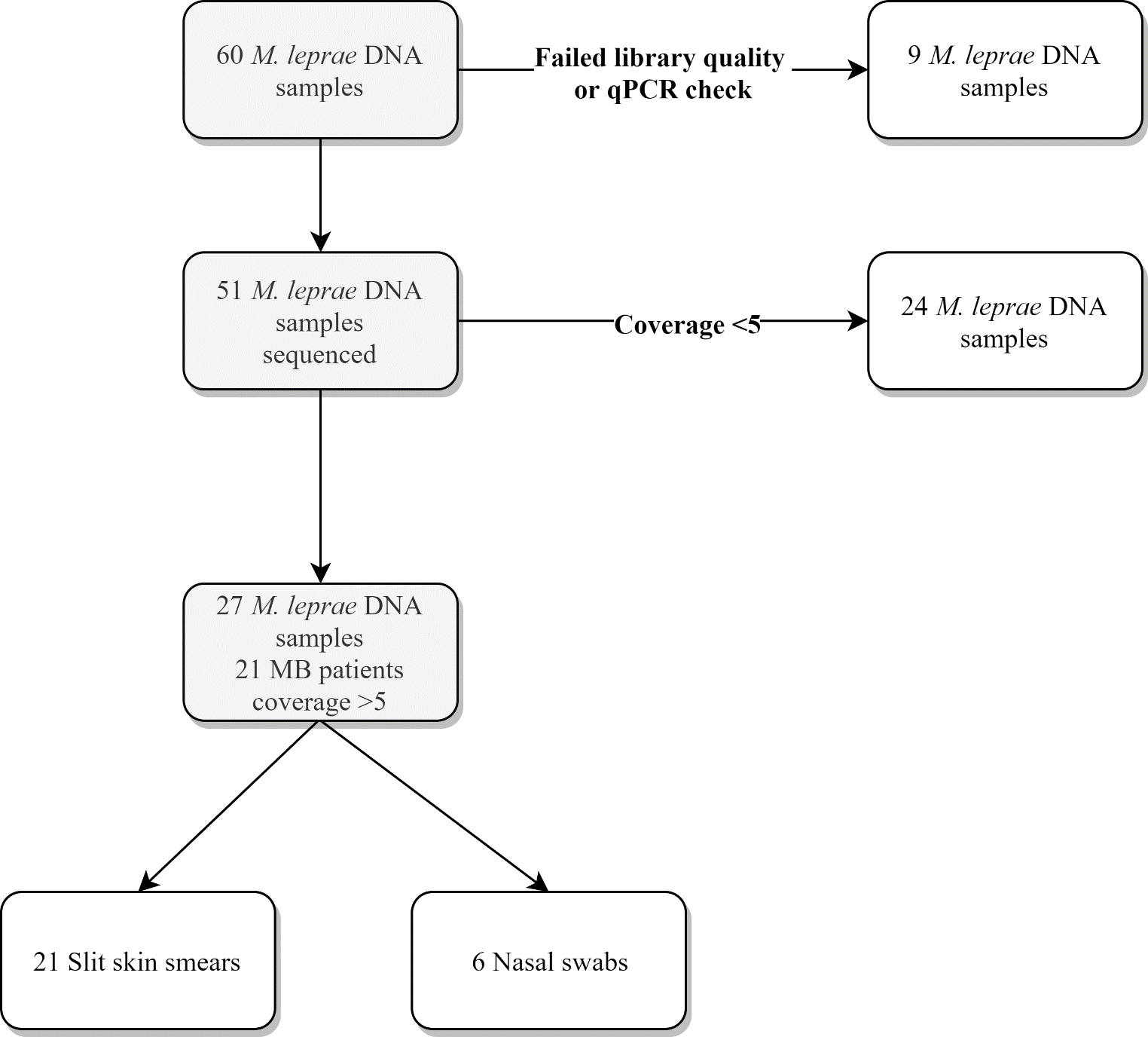
**

**Figure S1. Samples analysed by whole genome sequencing.** Number of DNA samples isolated from slit skin smears (SSS) or nasal swabs (NS) analysed by whole genome sequencing, samples that failed quality checks and samples with a query coverage higher or lower than 5 for *Mycobacterium leprae*. Origin of DNA samples sequenced with a coverage>5 is shown. All samples sequenced were collected from multibacillary (MB) patients. For all samples obtained from NS a sample of the same subject from SSS was also successfully sequenced.

**References**

Donoghue, H.D., Holton, J., and Spigelman, M. (2001). PCR primers that can detect low levels of *Mycobacterium leprae* DNA. *J Med Microbiol* 50(2)**,** 177-182. doi: 10.1099/0022-1317-50-2-177.

Martinez, A.N., Lahiri, R., Pittman, T.L., Scollard, D., Truman, R., Moraes, M.O., et al. (2009). Molecular determination of *Mycobacterium leprae* viability by use of real-time PCR. *J Clin Microbiol* 47(7)**,** 2124-2130. doi: 10.1128/jcm.00512-09.

Monot, M., Honore, N., Garnier, T., Zidane, N., Sherafi, D., Paniz-Mondolfi, A., et al. (2009). Comparative genomic and phylogeographic analysis of *Mycobacterium leprae*. *Nat Genet* 41(12)**,** 1282-1289. doi: 10.1038/ng.477.

Truman, R.W., Singh, P., Sharma, R., Busso, P., Rougemont, J., Paniz-Mondolfi, A., et al. (2011). Probable zoonotic leprosy in the Southern United States. *The New England journal of medicine* 364(17)**,** 1626-1633. doi: 10.1056/NEJMoa1010536.

WHO (2017). "A guide for surveillance of antimicrobial resistance in leprosy". (New Delhi: World Health Organization, Region Office for South-East Asia).
